# Proposal of New Safety Limits for In Vivo Experiments of Magnetic Hyperthermia Antitumor Therapy

**DOI:** 10.3390/cancers14133084

**Published:** 2022-06-23

**Authors:** Borja Herrero de la Parte, Irati Rodrigo, Jon Gutiérrez-Basoa, Sira Iturrizaga Correcher, Carmen Mar Medina, Jose Javier Echevarría-Uraga, Jose Angel Garcia, Fernando Plazaola, Ignacio García-Alonso

**Affiliations:** 1 Department of Surgery and Radiology and Physical Medicine, Faculty of Medicine and Nursing, University of the Basque Country UPV/EHU, ES48940 Leioa, Spain; ignacio.galonso@ehu.eus; 2 Interventional Radiology Research Group, Biocruces Bizkaia Health Research Institute, ES48903 Barakaldo, Spain; josejavier.echevarriauraga@osakidetza.eus (J.J.E.-U.); joseangel.garcia@ehu.eus (J.A.G.); fernando.plazaola@ehu.eus (F.P.); 3 Department of Bioengineering, 340 Hearst Memorial Mining Building, University of California, Berkeley, CA 94720, USA; 4 Department of Electricity and Electronics, Faculty of Science and Technology, University of the Basque Country UPV/EHU, ES48940 Leioa, Spain; 5 Department of Gastroenterology and Hepatology, General University Hospital Consortium of Valencia, ES46014 Valencia, Spain; jgutierrez060@ikasle.ehu.eus; 6 Department of Clinical Analyses, Galdakao-Usansolo Hospital, ES48960 Galdakao, Spain; sira.iturrizagacorrecher@osakidetza.eus (S.I.C.); mariadelcarmen.marmedina@osakidetza.eus (C.M.M.); 7 Department of Radiology, Galdakao-Usansolo Hospital, ES48960 Galdakao, Spain; 8 Department of Physics, Faculty of Science and Technology, University of The Basque Country UPV/EHU, ES48940 Leioa, Spain

**Keywords:** antitumor therapy, magnetic hyperthermia, alternating magnetic field, Atkinson–Brezovich limit, *H × f* product, biochemical changes, *H × f* safety limit

## Abstract

**Simple Summary:**

Magnetic hyperthermia is a promising therapy for the treatment of certain types of tumors. However, it is not clear what the maximum limit of the magnetic field to which the organism can be subjected without severe and/or irreversible pathophysiological consequences is. This study aims to study the alterations at the physiological level that may occur after exposure to different combinations of frequency and intensity of the applied alternating magnetic field. Understanding the response to alternating magnetic field exposure will allow us to apply this type of antitumor treatment in a safer way for the patient, while achieving an optimal therapeutic result.

**Abstract:**

Background: Lately, major advances in crucial aspects of magnetic hyperthermia (MH) therapy have been made (nanoparticle synthesis, biosafety, etc.). However, there is one key point still lacking improvement: the magnetic field-frequency product (*H × f* = 4.85 × 10^8^ Am^−1^s^−1^) proposed by Atkinson–Brezovich as a limit for MH therapies. Herein, we analyze both local and systemic physiological effects of overpassing this limit. Methods: Different combinations of field frequency and intensity exceeding the Atkinson–Brezovich limit (591–920 kHz, and 10.3–18 kA/m) have been applied for 21 min to WAG/RijHsd male rats, randomly distributed to groups of 12 animals; half of them were sacrificed after 12 h, and the others 10 days later. Biochemical serum analyses were performed to assess the general, hepatic, renal and/or pancreatic function. Results: MH raised liver temperature to 42.8 ± 0.4 °C. Although in five of the groups the exposure was relatively well tolerated, in the two of highest frequency (928 kHz) and intensity (18 kA/m), more than 50% of the animals died. A striking elevation in liver and systemic markers was observed after 12 h in the surviving animals, independently of the frequency and intensity used. Ten days later, liver markers were almost recovered in all of the animals. However, in those groups exposed to 591 kHz and 16 kA/m, and 700 kHz and 13.7 kA/m systemic markers remained altered. Conclusions: Exceeding the Atkinson–Brezovich limit up to 9.59 × 10^9^ Am^−1^s^−1^ seems to be safe, though further research is needed to understand the impact of intensity and/or frequency on physiological conditions following MH.

## 1. Introduction

Antitumor therapy based on the heating of pathological tissue, currently known as hyperthermia, is a therapeutic technique that has been known for several centuries, even millennia [1]. As early as 3000 B.C., an Egyptian papyrus records a technique for the control or treatment of a breast tumor by generating heat by rapidly rotating a stick directly on the tumor surface, like a “fire drill” [2], although it is true that, according to the description provided in the text, this procedure resembles what is currently known as thermo-ablation.

Aside from these ablative techniques, the first references to a type of hyperthermia similar to what we know today as whole body hyperthermia (WBH) appear at the end of the 18th and beginning of the 19th centuries, when several treatises described partial or total tumor remissions in patients with high fevers due to malaria or erysipelas [3]. Currently, the preferred hyperthermia procedures involve locally applied hyperthermia rather than WBH, largely due to its minor or minimal side effects on other organs. Among the various methods to induce localized hyperthermia (infrared, microwave, radiofrequency,…), in this work we opted for an electromagnetic heating method, by exposure to an alternating magnetic field (AMF) inductor, to achieve magnetic hyperthermia (MH).

MH is a novel treatment that is induced using magnetic nanoparticles (MNPs) to target cancer tissues by means of antibodies and applying an AMF of frequencies ranging between 100 kHz and 1 MHz, in the target area. Under such conditions, MNPs act as very local heat sources, which are capable of raising the temperature of cancer tissues and consequently induce apoptosis or necrosis of the tumor [4,5,6,7,8,9,10].

The first published study on locally applied and controlled MH was published in 1957 [11]. Gilchrist et al. infused Fe_2_O_3_ magnetic nanoparticles into lymph nodes to treat colon and rectal cancer metastases. They demonstrated that they successfully delivered nanoparticles to lymph nodes that likely contain metastases. Furthermore, in in vitro experiments, when the same nanoparticles (5 mg per gram of lymphatic tissue) were exposed to 16–19.2 kA/m at 1.2 MHz, they reported an increase of 14 °C in three minutes. However, since no control experiments were performed, to date it is not possible to determine whether the thermal increase was due to the heating of the nanoparticles or was directly related to the electric field generated, given the high field frequency used. Fifty years later, in 2010, MagForce AG company (Berlin, Germany) obtained European Union Regulatory Approval (10/2011) for its the Nanotherm^®^ therapy and later in 2013 started a clinical study in current glioblastoma with Nanotherm^®^ therapy at the Charité Hospital in Berlin. Recently, the FDA approved the Nanotherm^®^ therapy system for intermediate-risk prostate cancer [12,13].

In order to achieve an efficient MH treatment, MNPs should display the best heating properties and generate as much heat as possible, at the lowest particle content. Since the experiments carried out by Gilchrist et al. in 1957, nanoparticle delivery techniques and AMF generation devices, have greatly improved, and even changes in nanoparticles synthesis, have enhanced their thermal properties. However, the only parameter that remains apparently unchanged is the safety conditions established by the *H × f* product, known as the Atkinson–Brezovich limit [14,15]. Defining a correct safety limit became crucial to maintain patient safety while maximizing MNPs heating. In the last decades, different safety limits have been proposed, however, there is still an ongoing discussion on this topic.

As described by Faraday’s law, an AMF passing through a conductor material induces an electrical current, called eddy currents. In clinical application of MH, the body tissues are conductors, so eddy currents are induced in the patient when subjected to the alternating magnetic field. Eddy currents can produce a non- specific heating in the body that can affect negatively healthy tissues of humans. However, there is a patient tolerable limit, known as safety limit, below which the eddy current effects are bearable.

Atkinson et al., in 1984 [14], and later Brezovich et al., in 1988 [15], studied the effect that the eddy currents can have on humans. They estimated that the rate of heat production per unit of tissue volume for a cylindrical body is:

(1) $$P=\sigma {\left(\pi \mu_{0}\right)}^{2}{\left(H\cdot f\right)}^{2}r^{2}\;,$$

where σ is the electrical conductivity of the tissue, $\mu_{0}$ is the vacuum permeability, the radio (r) of the cylinder and *H* and *f* the magnetic field intensity and frequency, respectively.

In these experiments, some clinical tests were performed using a 30 cm diameter single turn coil placed around the torso of an average healthy patients. It was found that at a frequency of 13.56 MHz, patients could tolerate magnetic field intensities up to 36.3 A/m for extended time periods without considerable discomfort. Based on this study, the highest acceptable value was established to be *H × f* = 4.85 × 10^8^ Am^−1^s^−1^. However, as heat production depends on the size the exposed tissue, the Atkinson–Brezovich criterion should not be considered as the only criterion. For smaller a diameter of the body region under exposure, a higher *H × f* safety limit would be permissible. In Nanotherm^®^ therapy, patients with glioblastoma can tolerate magnetic fields up to 18 kA/m at 100 kHz (1.8 × 10^9^ Am^−1^s^−1^) [16,17,18]. This increase in the safety limit arises from the diameter difference between the head of a patient with glioblastoma and the torso in Atkinson et al. So, for considering smaller diameter of the exposed body region, Hergt et al., suggested a weaker criterion of 5 × 10^9^ Am^−1^s^−1^, that is, one order of magnitude bigger than the Atkinson–Brezovich safety limit [19]. It should be noted that no experimental tolerance test has been repeated since 1984. Therefore, it is essential to carry out new research in this area, not only to find a new safety limit, but also to study the implication of the magnetic field and the frequency in the product *H × f*.

In this work, we present an in vivo magnetic hyperthermia tolerance test carried out at a magnetic field intensity up 18 kA/m and frequencies between 594 kHz and 928 MHz, and new safety limit is proposed, based on biochemical parameters that analyze the systemic response to AMF.

## 2. Materials and Methods

### 2.1. Magnetic Hyperthermia Device

**Figure 1:**
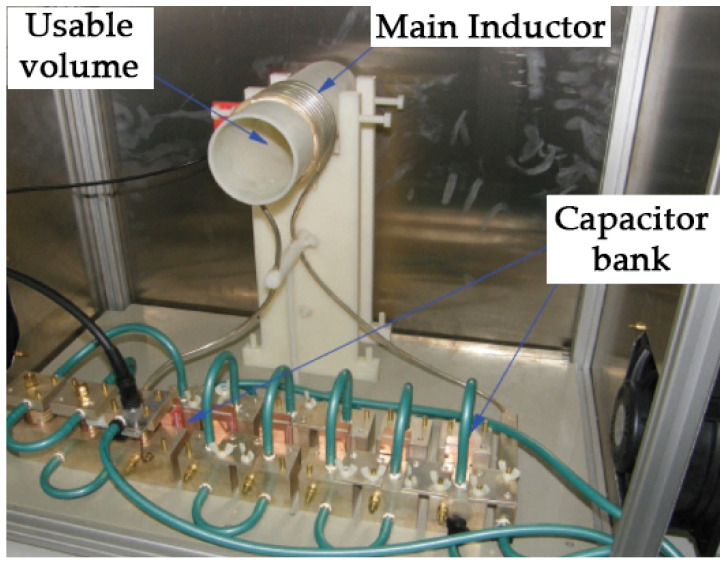
Alternating magnetic field (AMF) inductor. The picture shows its main components.

Exposures to AMF were performed using a home-made electromagnet previously described by our group [20,21]. The set-up consisted of a solenoid coil 8 cm long, 6 cm in diameter and 9 turns that generated a homogeneous AMF within a volume inside the coil, where the animals were placed (Figure 1). The sinusoidal signal to drive the system was produced by a signal generator (model 33220A, Agilent, Santa Clara, CA, USA) and amplified by a linear power amplifier of 2 kW maximum power (model 1240L Broadband Power Amplifier, Electronics and Innovation Ltd., Rochester, NY, USA). The magnetic field generated by the solenoid coil was measured during the experiments using an external coil.

### 2.2. In Vivo Magnetic Hyperthermia Therapy

All the procedures were performed in accordance with current national legislation on animal experimentation and were approved by the Internal Review Board of the University of the Basque Country (UPV/EHU) (ref.: M20/2018/028 Herrero de la Parte).

The study was carried out on 90 WAG/RijHsd male rats, three months old and weighing between 260 and 290 g. The animals had free access to water and a standard laboratory diet; animal house humidity and temperature were kept constant in accordance with current regulations and a 12-h light/dark cycle was established.

The rats were randomly distributed in seven experimental groups (G1–G7; twelve animals in each group) that were exposed to an AMF of different intensity and frequency. Half of the animals (six) in each experimental group were sacrificed after 12 h of exposure to AMF, while the other half were sacrificed after 10 days; this approach was aimed to check the immediate or acute damage (12 h) and the medium–long term or chronic damage (10 days). Additionally, another six animals that did not undergo AMF, nor any surgical procedure, were used as a control group (G0).

AMF exposure procedures were in accordance with those previously published by our research group [20,21]. Briefly, once the animals were properly anesthetized (intraperitoneal injection of diazepam, ketamine and medetomidine), a middle laparotomy was performed to expose the liver, and two non-resistive fiber optic thermometers OTG-MTK5 (Opsens, Québec, QC, Canada) were located in the animals in order to monitor thermal changes. One of them was placed between the left lateral and paramedian liver lobes, while the other one was placed within the rectum. Once the thermal probes were set, the animal was positioned inside the solenoid coil and the treatment was applied. In accordance with our previously reported experience, magnetic hyperthermia treatments were performed for 21 min, in the magnetic field shown in Table 1 in the described magnetic hyperthermia configuration.

To ensure that the animals did not overheat, once the thermal probe placed in the liver reached 43 °C, the software controlling the AMF generator automatically adapted the field intensity using an on/off controller.

### 2.3. Blood and Tissue Collection

As stated, the effects of exposure to the different magnetic fields were tested after 12 h or 10 days (according to the experimental group). This required the extraction of both blood samples to obtain serum samples and liver tissue samples for histological analysis. Samples were obtained under 1.5% isoflurane anesthesia; first, the entire volume of circulating blood (5–6 mL) was collected from the inferior cava vein and immediately centrifuged for 10 min at 3000 rpm to separate the serum. Once the serum was obtained, it was frozen until subsequent analysis.

### 2.4. Biochemical Analysis

The functional state of the liver, kidneys and pancreas, as well as the general status of the organism after hyperthermia, was evaluated by quantifying serum enzymes such as alanine transaminase (ALT), aspartate transaminase (AST), creatinine (Cr), amylase, alkaline phosphatase (ALP), creatine kinase (CK), and lactate dehydrogenase (LDH).

These analyses were performed on a Cobas^®^ 8000 modular clinical analyzer, equipped with a Cobas c702 module (Hoffmann-La Roche, Basel, Basel-Stadt, Switzerland), and kits for enzymes’ quantification (Roche Diagnostics GMBH, Rotkreuz, Zug, Switzerland).

### 2.5. Statistical Analysis

The quantitative variables described throughout the study were presented as mean and standard deviation, once the Kolmogorov–Smirnov normality test had been passed. Statistical analysis of the data was performed by analysis of variance (ANOVA) using GraphPad Prism software (v8.2.1, GraphPad Software, San Diego, CA, USA), accepting a minimum level of significance of *p* < 0.05. If statistically significant differences were found between the experimental groups, multiple comparison tests were also carried out (Tukey’s multiple comparison test for between-groups comparison).

## 3. Results

### 3.1. In Vivo Hyperthermia

After exposure to the AMF inducer, mortality following the procedure was the first of the indicators analyzed. The survival rate of the rats is shown in Table 1. It could not confirm that the same *H × f* values were directly correlated to survival rate; as can be seen, at the highest values of intensity (G2) and frequency (G7) were the lowest survival rates. In such extreme cases all animals died within 4 h after the end of exposure to AMF. In the G2 group, the four animals that survived longer than the initial 4 h were subsequently euthanized due to their clinical conditions, in adherence to animal welfare standards and in accordance with the 3Rs principle.

Another three animals died after hyperthermia treatment, one in the G5 group and two in the G6 group; the remaining animals that completed the whole study showed no clinical signs that would indicate any type of post-treatment complication.

**Figure 2:**
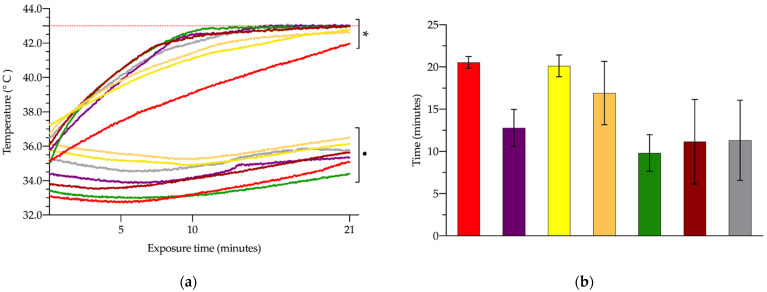
Absolute temperature values (**a**) registered in the liver (asterisk) and rectal probe (dot), after exposure to alternating magnetic field (AMF), with different combinations of intensity and frequency: 14 kA/m and 591 kHz (G1, red); 18 kA/m and 591 kHz (G2, purple); 16 kA/m and 591 kHz (G3, yellow); 13.7 kA/m and 700 kHz (G4, orange); 14.4 kA/m and 774 kHz (G5, green); 11.2 kA/m and 856 kHz (G6, garnet), and 10.3 kA/m and 928 kHz (G7, grey); the upper dotted orange line indicates the upper temperature limit set at 43 °C for the hepatic probe, the threshold temperature. Required time to reach the 43 °C limit in the hepatic parenchyma (**b**).

Figure 2a and Table 2 show the evolution and absolute values of hepatic and rectal temperature recorded for each group throughout the exposure period. As can be seen, most of the experimental groups reached the maximum temperature, limited to 43 °C, with the exception of the G1 group, which did not exceed 42 °C. In contrast, rectal temperature remained almost the same, with no statistically significant changes between the different experimental groups (*p* > 0.05).

Figure 2b illustrates how long it took for each experimental group to reach the 43 °C self-imposed limit of 43 °C on liver temperature. Groups G5, G6 and G7 reached 43 °C after just 10–11 min, with no significant differences between these three groups. The other three groups, G2, G3, and G4, reached the limit a few minutes later (*p* < 0.05), that is, 13 ± 2.2, 20 ± 1.3, and 17 ± 3.8 min respectively (*p* < 0.05).

**Figure 3:**
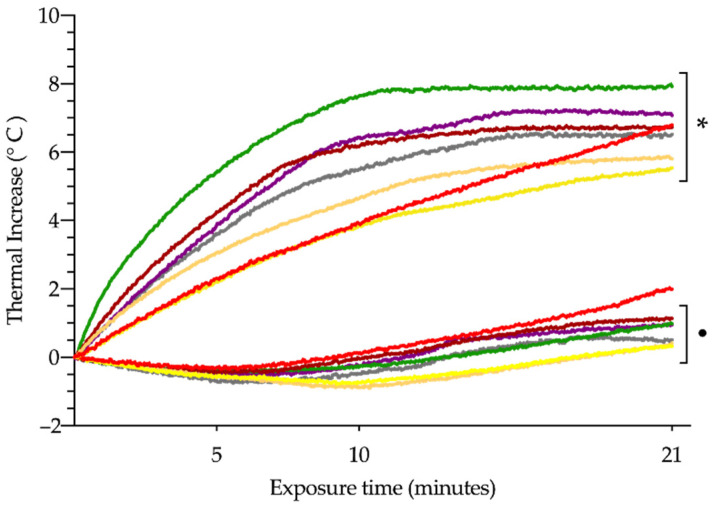
Thermal increase in both liver tissue (asterisk) and rectum (dot) after exposure to alternating magnetic field (AMF), with different combinations of intensity and frequency: 14 kA/m and 591 kHz (G1, red); 18 kA/m and 591 kHz (G2, purple); 16 kA/m and 591 kHz (G3, yellow); 13.7 kA/m and 700 kHz (G4, orange); 14.4 kA/m and 774 kHz (G5, green); 11.2 kA/m and 856 kHz (G6, garnet), and 10.3 k kA/m and 928 kHz (G7, grey).

Considering the thermal increase throughout the exposure time (Figure 3), we observed that the overall mean increase in hepatic and rectal temperatures was 6.63 °C and 0.90 °C, respectively. The liver thermal increase did not present statistically significant differences amongst the different experimental groups, whereas the thermal increase of the rectum showed significant differences between G1 and G3 (1.99 ± 1.46 vs. 0.33 ± 0.79 °C, respectively; *p* < 0.05) and G1 and G4 (1.99 ± 1.46 vs. 0.42 ± 0.76 °C, respectively; *p* < 0.05).

### 3.2. Biochemical Changes after Exposure to AMF

In general terms, the serum levels of the enzymes analyzed showed a similar trend; resulting in an increase in those serum samples analyzed 12 h after exposure to AMF, followed by a trend to recover normal values after 10 days, although not all the indicators analyzed were able to reach baseline levels. Biochemical data could not be obtained for groups G2 and G7 due to the death of the animals, either by death after exposure to AMF or by sacrifice in accordance with humane endpoint criteria.

First, the enzymatic alterations in each group were studied in relation to the parameters obtained from the control animals (Table 3); subsequently, the intergroup variations were analyzed separately, twelve hours and ten days later, to determine whether the alterations in frequency and intensity, while fixing the *H × f* product, modified the analytical parameters of the blood tests.

**Figure 4:**
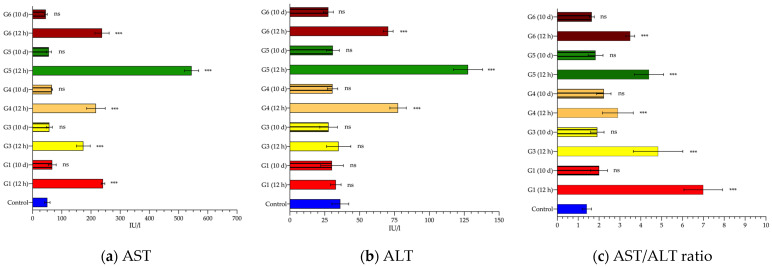
Serum values of liver enzymes. Aspartate transaminase (AST) (**a**), alanine transaminase (ALT) (**b**), and AST/ALT ratio (De Ritis ratio) (**c**) mean values (and standard deviation) measured in blood samples collected 12 h (smooth pattern) or 10 days (striped pattern) from control (blue), or alternating magnetic field (AMF)-exposed rats: 14 kA/m and 591 kHz (G1, red); 16 kA/m and 591 kHz (G3, yellow); 13.7 kA/m and 700 kHz (G4, orange); 14.4 kA/m and 774 kHz (G5, green); 11.2 kA/m and 856 kHz (G6, garnet). Values are expressed in international units per liter (IU/L). Statistical significance is shown compared to the control group values (***: *p* < 0.001; ns: *p* > 0.05).

Regarding liver enzymes, AST (Figure 4a and Table 3), after 12 h, was significantly elevated in all experimental groups, ranging from 3.4-fold (group G3) to 10.7-fold (group G5) compared to the control group (241 ± 6.2 IU/L). Ten days later, the plasma levels registered in all animals returned to baseline values, with no significant differences with respect to the control (51 ± 9.3 UI/L). A similar finding was noted for ALT (Figure 4b and Table 3). However, in this case, a significant change in ALT levels was only evident in G4, G5 and G6 groups after 12 h; in groups G1 and G3, the values remained within the normal range (36 ± 6.1 IU/L). After 10 days, there was no evidence of any disturbance in blood concentrations.

When the AST/ALT ratio (also called De Ritis ratio) was calculated, the same pattern was again observed (Figure 4c and Table 3). The ratio in the control group was 1.4 ± 0.22. This figure was considerably higher for the groups analyzed after 12 h (between 2 to 5 times; *p* < 0.001); however, when the ratio of the enzymes quantified ten days later was calculated, no statistically significant differences were found, although it is true that the values were slightly elevated (1.2 to 1.6 times; *p* > 0.05) with respect to the control (1.4 ± 0.2).

Considering only those groups exposed to AMF, at 12 h, we clearly distinguished two patterns in the observed elevations in transaminases (Figure 4a,b), especially ALT. It was particularly marked for ALT, showing average values up to three times higher in the groups exposed to the highest frequencies, between 700 and 928 kHz, compared to groups G1 and G3 (591 kHz, and 14 and 16 k/Am, respectively).

**Figure 5:**
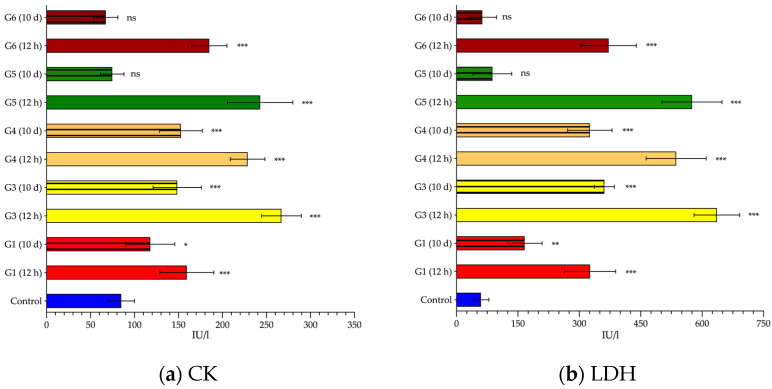
Serum values of ubiquitous enzymes. Creatine kinase (CK) (**a**), lactate dehydrogenase (LDH) (**b**) mean values (and standard deviation) measured in blood samples collected 12 h (smooth pattern) or 10 days (striped pattern) from control (blue) or alternating magnetic field (AMF)-exposed rats: 14 kA/m and 591 kHz (G1, red); 16 kA/m and 591 kHz (G3, yellow); 13.7 kA/m and 700 kHz (G4, orange); 14.4 kA/m and 774 kHz (G5, green); 11.2 kA/m and 856 kHz (G6, garnet). Values are expressed in international units per liter (IU/L). Statistical significance is shown compared to the control group values (*: *p* < 0.05; **: *p* < 0.01; ***: *p* < 0.001; ns: *p* > 0.05).

Analyzing more ubiquitous enzymes, not specific to any particular tissue, such as CK (Figure 5a and Table 3) and LDH (Figure 5b and Table 3), we can clearly appreciate how, once again, the alteration in their levels was similar to previous findings; there was a sudden rise in their levels at 12 h, tending to normalize after 10 days, but not completely returning to normal. In these cases, we could not clearly identify whether CK and/or LDH were influenced by either factor (frequency or intensity) independently. It can only be observed that the combination of frequency and intensity that had the least systemic impact was G1 (591 kHz and 14 kA/m). For the other groups, the average values of CK and LDH detected were 1.5 and 1.6 times higher, respectively, compared to the G1 group.

**Figure 6:**
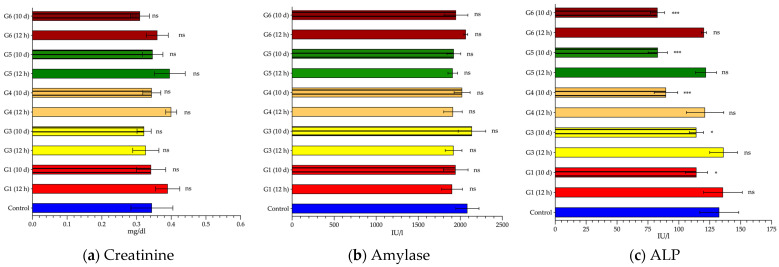
Serum enzyme values specific to other systems and/or organs. Creatinine (**a**), amylase (**b**), and alkaline phosphatase (ALP) (**c**) mean values (and standard deviation) measured in blood samples collected 12 h (smooth pattern) or 10 days (striped pattern) from control (blue) or alternating magnetic field (AMF)-exposed rats: 14 kA/m and 591 kHz (G1, red); 16 kA/m and 591 kHz (G3, yellow); 13.7 kA/m and 700 kHz (G4, orange); 14.4 kA/m and 774 kHz (G5, green); 11.2 kA/m and 856 kHz (G6, garnet). For creatinine, values are expressed in milligrams per deciliter (mg/dL), and both for amylase and ALP, figures are expressed in international units per liter (IU/L). Statistical significance is shown compared to the control group values (*: *p* < 0.05; ***: *p* < 0.001; ns: *p* > 0.05).

Finally, creatinine, amylase and ALP did not add relevant information to our study. Neither creatinine (Figure 6a) nor amylase (Figure 6b) suffered, in general, major changes in the different experimental groups. Measured levels of creatinine (0.31 to 0.40 mg/dL) were within the normal range and detected amylase concentrations ranged from 1903 to 2140 IU/L; differences between groups were not significant for neither enzyme. The response of ALP (Figure 6c) was completely different. Twelve hours after AMF-exposure, ALP remained similar to baseline concentrations, but after 10 days there was a significant decrease in all groups (*p* < 0.05).

## 4. Discussion

In the last two decades, some groups have proposed the redefinition of the Atkinson–Brezovich limit. Some of them, such as Hergt et al. [19], proposed to raise it. They estimated that for the exposure of small body regions this limit can be exceeded up to 10 times, up to *H × f* ≤ 5 × 10^9^ Am^−1^s^−1^. In contrast, authors such as Dossel et al. suggested an even more restrictive limit. In a model of a human body, they simulated the heat resulting from the distribution of AMF and proposed to halve the Atkinson–Brezovich limit, 2 × 108 Am^−1^s^−1^ at most [22]. However, both Hergt’s and Döseel’s limits were proposed only on the basis of theoretical or phantom studies; neither was tested on a living organism to investigate the systemic pathophysiological response. Indeed, Atkinson and Brezovich also failed to analyze this issue. Their limit (4.85 × 108 Am^−1^s^−1^) was established only considering whether the patient was able to withstand one hour of treatment without referring any major discomfort [15].

In contrast, other researchers did carry out in vivo studies surpassing the Atkinson–Brezovich limit [23,24,25,26]. Kossatz and colleagues [23], in a xenograft model induced by injection of MDA-MB-231 cells (human breast adenocarcinoma), raised the *H × f* value 10 times by exposing the animals to an AMF of 15.4 kA/m and 435 kHz (6.72 × 10^9^ Am^−1^s^−1^). Another two reports led by Thiesen [24] and Jordan [25], in rats bearing gliomas, demonstrated the therapeutic effectiveness of AMF (0–18 kA/m, 100 kHz; 1.9 × 10^9^ Am^−1^s^−1^), raising the tumor temperature to 43–47 °C during a 40-min period. Finally, even more aggressive in their approach were Lee et al. [26], as they raised the Atkinson–Brezovich limit up to 100 times to treat nude mice xenografted with human malignant glioma (U87MG cancer cells) (37.3 kA/m and 500 kHz; 1.87 × 10^10^ Am^−1^s^−1^). Despite the fact that they have tested higher AMFs in vivo than Atkinson and Brezovich, all of them only focused their attention on analyzing the therapeutic benefit, not the pathophysiological implications of whole body AMF exposure.

One of the main shortcomings found in the reviewed literature, regarding the application of MH, is the lack of concern or inexistence of studies that analyze the biosafety of exposure to AMF above the Atkinson–Brezovich limit. However, many studies have evaluated the cytotoxicity or biosafety of the administration of magnetic nanoparticles [27,28,29,30,31]. To the best of our knowledge, our study is the first reported investigation that attempts to reestablish or redefine a new limit of the *H × f* product for in vivo MH experiments, based on biochemical parameters that analyze the systemic response to MH. 

The proposed combinations of magnetic field intensity and frequency used in this study were established on the basis of previously published work by our own research group in 2016 [20] and 2021 [21]. In those publications, we demonstrated both the therapeutic effectiveness and the non-harmfulness of the procedure; an exposure of twenty-one minutes at 606 kHz and 14 kA/m (8.48 × 10^9^ Am^−1^s^−1^) induced a 30% destruction of the tumor mass [20], while not leading to irreversible alterations in the biochemical profile and/or metabolomic profile of the animals [21]. Due to the modifications implemented in our homemade AMF device used in those previous studies, in the present study it was not possible to adopt exactly the same combination of frequency and intensity, given the new equipment configuration, but rather the most similar combination possible was used (591 kHz and 14 kA/m; 8.27 × 10^9^ Am^−1^s^−1^).

Because of the shortage of evidence in the literature on biochemical changes due to hyperthermia, we have compared and discussed much of our results with the existing literature on heat stroke (HS), defined as “the rise in core body temperature when heat accumulation overrides heat dissipation during exercise or exposure to environmental heat stress” [32].

Pan et al. also treated liver tumor implants with MH [33]. They applied 1.7 mT AMF (1.35 kA/m, and 577 kHz; 7.79 × 10^8^ Am^−1^s^−1^) for 20 min, reaching 44 °C in the tumor tissue. After 28 consecutives days of treatment, biochemical analysis (AST, ALT, TP, TBil, albumin, and uric acid) did not show significant changes compared to control values. This recently published paper supports our hypothesis that the Atkinson–Brezovich limit can be safely exceeded; in fact, Pan et al. doubled the *H × f* product in their experiments.

Mustafa et al. subjected rabbits to WBH, placing them in chambers at 43 °C, and analyzed its effects on renal function, assessing, among others, creatinine levels [34]. They demonstrated an inverse relationship between renal function, and the creatinine levels and temperature increase in rabbits. Renal function decreased, resulting in increased serum creatinine levels, as rabbit body temperature (measured in the rectum) increased by 2, 3, or 4 °C. However, in our study, no significant changes in creatinine were found, which indicates that renal function is not affected in our model of MH. A feasible explanation for this dissimilarity of results between Mustafa’s work and our work could be the difference in the methods used in the application of hyperthermia. While Mustafa et al. applied WBH, raising body temperature by 2 to 4 °C, we did not significantly increase the body temperature, as can be seen in Figure 2a and Table 2.

First of all, it is important to point out that the alterations in any of the analyzed enzymes do not arise solely and exclusively from damage to one organ or system, but rather all of them are in a complex balance during the normal functioning of the organism. It is clear that our MH set up provokes both liver tissue alteration and a nonspecific systemic response (Figure 4 and Figure 5). Although it is true that the systemic response observed (elevation of CK and LDH) could be due to the surgical procedure performed to place the thermal probe in the liver [35,36,37], we cannot rule out that this elevation may also be exacerbated by exposure to AMF.

It is well-known that HS triggers major changes in blood biochemical parameters, systemic inflammation, and coagulopathy [38,39,40]. Following HS, many intracellular components were released into the blood flow; among others, high levels of CK, LDH and AST are commonly detected in patients with HS [41,42]. Under physiological conditions, these biomarkers are contained within the cytoplasm of muscle cell; thus, they are widely used to determine muscle damage, also called rhabdomyolysis, because of their release into the bloodstream correlates with muscle fatigue and/or damage [43,44,45,46]. Therefore, based on these supporting data, particularly in the context of MH, the elevation that we were able to detect in CK, LDH, and AST seems to be consistent with the rhabdomyolysis phenomena associated with HS. However, the elevation of AST is not only due to the aforementioned damage by rhabdomyolysis. Instead, since it is a ubiquitous enzyme, many other clinical alterations can induce its increase, such as liver injury. In this context, ALT was also seen to be elevated. Zhang et al. also observed of ALT and AST elevation (more than 4 times) when the rats were subjected to HS (established by mean arterial pressure <25 mmHg and rectal temperature >42 °C) [47]. Histological evaluation of the liver slices showed severe sinusoidal congestion, associated with the presence of microthrombi around the sinusoidal area, as well as infiltration of inflammatory cells, and some foci of hepatocyte necrosis. In addition, markers induced by HS (thrombomodulin, nitric oxide, endothelin 1, hyaluronic acid), and related to inflammatory lesions (intercellular adhesion molecule-1 (ICAM-1), TF, and IL-6), were also dramatically affected. They concluded that, in HS, the onset of the liver injury may be triggered by morphological and functional changes in the sinusoidal endothelial cells; such changes may even worsen liver damage due to the proinflammatory, proadhesive, and procoagulant effects exhibited by the disrupted sinusoidal endothelial cells.

Li et al., in a model of exertional HS under high temperature and humidity conditions, also evidenced liver damage due to high temperatures [48]. Electron microscopy analysis revealed that the normal appearance of liver cells had disappeared, showing loss of cellular organelle and mitochondrial structure. In addition, AST values and de Ritis ratio (AST/ALT) were enhanced in high temperature and high humidity exercising group. Inflammatory response markers (IL-6 and TNF-α) and a product of DNA oxidation under oxidative stress (8-OhdG) were also enhanced. All these findings suggest the occurrence of liver cell apoptosis.

Another tricky issue when analyzing biochemical alterations is the limited information regarding their evolution in the medium and long term. Ward et al. conducted a retrospective study of HS patients records of US military personnel [49]. They found sings of acute liver damage and myiolisis; however, the figures showed a trend towards normal values after the third to fifth day, reaching the normal range after the seventh day. ALT still remained above the normal range at the end of the study, on day 14. This outcome shows the same trend as observed in our results. The initial thermal shock causes a marked rise in the parameters that, over time, tends to normalize. This fact, as also reported by Ward et al., demonstrates that the organism can tolerate thermal exposure of up to 43 °C with no severe or irreversible side effects.

After these remarks, before proposing a new maximum *H × f* product, we must consider how the variation in frequency and/or intensity has induced different responses at the pathophysiological level. In our piece of work, in terms of survival from exposure to magnetic fields, it is clear that those fields with higher frequencies (774 kHz, 856 kHz and 928 kHz) or with an intensity of 18 kA/m are the most harmful. Slight differences in the *H × f* product estimated for groups G3 to G7 (from 9.46 × 10^9^ Am^−1^s^−1^ to 9.59 × 10^9^ Am^−1^s^−1^) do not seem to exert a striking impact on the results obtained.

However, when excluding those groups (G2 and G7) where the mortality rate exceeded 50%, there is no clear association between high frequencies (>700 kHz) and high biochemical liver damage, and even less, non-recoverable damage. However, there is a persistent damage (non-recoverable after 10 days), in the markers of systemic damage (CK and LDH) in the groups with the lowest applied frequencies (591 kHz and 700 kHz). As previously mentioned, there is no evidence of damage to other organs, which may be due to the fact that the generated field is very close to the region of interest, the liver in our case. To the best of our knowledge, we have not been able to find a plausible explanation for these results in the published literature. We suggest that a possible hypothesis, or explanation, is that the different density of the tissues, their different conductivities and impedances, or their different percentage of water and ions, influences the generation of eddy currents that lead to the lethal and/or irreversible damage. This point should be tested in future research, perhaps in ex vivo models and not in animals, to look at how variations in water, density or ions alter their response to AMFs; unfortunately, this type of study has been carried out at frequencies an order of magnitude higher, analyzing the effect of MRI equipment [50]. Furthermore, we should not overlook that the proposed frequency and intensity should be able to induce sufficient heating of the nanoparticles needed in magnetic hyperthermia. With all of this, and considering the results presented here, it will be possible to formulate or propose new maximum and ideal conditions for experimentation in this area of knowledge.

Finally, it is important to note some of the shortcomings of this work that should be kept in mind when attempting to continue experimental research in this field, or even translate these results into a clinical setting.

Firstly, although it has been shown to be clinically safe to exceed the Atkinson–Brezovich limit, even above the *H × f* product used in another more recent work [23,24,25], it is important to point out that all the experiments were performed in healthy animals (non-bearing liver implants animals) and not previously subjected to surgery. This is not a minor concern, since bearing tumor implants, or previous surgery, alters and/or weakens the initial state of the patient, which could result in the patient not being able to successfully cope with the stress resulting from the heat shock due to exposure to the AMF. In addition, it must also be taken into account that the administration of a magnetofluid is necessary for nanoparticle-based hyperthermia therapy. Although many studies have shown that, with the correct preparation, these compounds do not, by themselves, produce a systemic reaction, the impact or response when all elements of this therapy are used in conjunction is not yet known. Lastly, and especially considering the intended extrapolation to human clinical practice, extreme caution must be exercised in the definition of the *H × f* limit. Our study and those previously referred to were carried out in small experimental animals (rodents). The diameter of the AMF inductor coil plays an important role in the eddy currents generated within the organism during exposure to AMF. It should be taken into account that, in our case, the diameter of the coil used was barely 6 cm, whereas Atkinson and Brezovich used coils with a diameter of 30 cm.

## 5. Conclusions

This study has demonstrated, that, from the physiological and animal recovery point of view, surpassing the Atkinson–Brezovich limit up to 9.46 × 10^9^ Am^−1^s^−1^ is possible and safe. However, given the multiple variables involved in establishing an appropriate combination of frequency and intensity of the applied magnetic field, more research is needed.

## Figures and Tables

**Table 1:** Applied electromagnetic fields according to the experimental group and survival rate of each of them.

Group Name	*f* (kHz)	*H* (kAm^−1^)	*H × f* (10^9^ Am^−1^s^−1^)	Survival Rate (% and Number)
G1	591	14	8.27	100% (12/12)
G2	591	18	10.64	33% (4/12)
G3	591	16	9.46	100% (12/12)
G4	700	13.7	9.59	100% (12/12)
G5	774	12.4	9.59	92% (11/12)
G6	856	11.2	9.59	83% (10/12)
G7	928	10.3	9.56	0% (0/12)

**Table 2:** Hepatic and rectal temperatures recorded at the onset (T_0_) and at the end (T_21_) of exposure to alternating magnetic fields, and the thermal increase in both tissues.

Group	T_0_ Hepatic Temperature	T_21_ Hepatic Temperature	Hepatic Thermal Increase	T_0_ Rectal Temperature	T_21_ Rectal Temperature	Rectal Thermal Increase
G1	35.2 ± 1.2 °C	42.0 ± 1.1 °C	6.8 ± 0.7 °C	33.1± 0.6 °C	35.1 ± 1.9 °C	2.0 ± 1.5 °C
G2	35.8 ± 0.3 °C	42.9 ± 0.1 °C	7.1 ± 0.3 °C	34.4 ± 0.6 °C	35.4 ± 0.8 °C	0.9 ± 0.9 °C
G3	37.3 ± 1.1 °C	42.8 ± 0.4 °C	5.5 ± 1.4 °C	35.8 ± 1.3 °C	36.1 ± 1.8 °C	0.3 ± 0.8 °C
G4	36.8 ± 0.9 °C	42.6 ± 0.6 °C	5.8 ± 1.0 °C	36.1 ± 0.9 °C	36.6 ± 1.2 °C	0.4 ± 0.8 °C
G5	35.0 ± 1.2 °C	43.0 ± 0.1 °C	7.9 ± 1.2 °C	33.4 ± 1.1 °C	34.4 ± 1.2 °C	1.0 ± 0.6 °C
G6	36.1 ± 0.5 °C	43.0 ± 0.1 °C	6.7 ± 1.0 °C	33.8 ± 0.9 °C	35.6 ± 0.8 °C	1.1 ± 1.1 °C
G7	35.0 ± 1.2 °C	43.0 ± 0.1 °C	6.5 ± 1.3 °C	35.3 ± 0.9 °C	35.8 ± 0.9 °C	0.5 ± 1.1 °C

**Table 3:** Serum levels of the enzymes analyzed: aspartate transaminase (AST), alanine transaminase (ALT), creatinine, amylase, creatine kinase (CK), lactate dehydrogenase (LDH), and alkaline phosphatase (ALP). The units of measurement for each parameter analyzed are indicated in parentheses: international units/liter (IU/L) or milligrams/deciliter (mg/dL). The *p* value indicates either the statistical significance of each experimental group compared to the control, or the non-existence of statistically significant differences (ns).

	Control	G1		G3		G4		G5		G6	
		12 h	10 Days	12 h	10 Days	12 h	10 Days	12 h	10 Days	12 h	10 Days
AST (IU/L)	51 ± 9.3	241 ± 6.2	67 ± 14	174 ± 23	58 ± 10	217 ± 32	66 ± 2.1	545 ± 24	56 ± 8.8	238 ± 24	45 ± 5
*p* value		<0.001	ns	<0.001	ns	<0.001	ns	<0.001	ns	<0.001	ns
ALT (IU/L)	36 ± 6.1	33 ± 3.7	30 ± 8.2	35 ± 8.7	28 ± 6.3	78 ± 5.9	31 ± 3.7	128 ± 10	31 ± 4.6	71 ± 3.5	28 ± 3.6
*p* value		ns	ns	ns	ns	<0.001	ns	<0.001	ns	<0.001	ns
AST/ALT ratio	1.4 ± 0.2	7 ± 0.9	2 ± 0.4	4.8 ± 1.2	1.9 ± 0.3	2.9 ± 0.7	2.2 ± 0.3	4.4 ± 0.7	1.8 ± 0.4	3.5 ± 0.2	1.7 ± 0.1
*p* value		<0.001	ns	<0.001	ns	<0.001	ns	<0.001	ns	<0.001	ns
Creatinine (mg/dL)	0.34 ± 0.06	0.39 ± 0.04	0.34 ± 0.04	0.33 ± 0.04	0.32 ± 0.02	0.40 ± 0.02	0.34 ± 0.03	0.40 ± 0.03	0.35 ± 0.03	0.36 ± 0.03	0.31 ± 0.03
*p* value		ns	ns	ns	ns	<0.01	ns	<0.05	ns	ns	ns
Amylase	2085 ± 138	1903 ± 123	1945 ± 148	1921 ± 99	2140 ± 163	1914 ± 110	2023 ± 95	1911 ± 57	1924 ± 57	2067 ± 23	1950 ± 143
*p* value		<0.001	ns	ns	ns	<0.001	ns	ns	ns	ns	ns
CK (IU/L)	85 ± 15	159 ± 31	118 ± 28	267 ± 22	149 ± 27	229 ± 20	153 ± 24	243 ± 37	75 ± 13	185 ± 20	67 ± 14
*p* value		<0.001	<0.05	<0.001	<0.001	<0.001	<0.001	<0.001	ns	<0.001	ns
LDH (IU/L)	60 ± 20	326 ± 63	167 ± 43	636 ± 56	361 ± 24	537 ± 73	326 ± 54	575 ± 73	88 ±+ 47	372 ± 68	63 ± 35
*p* value		<0.001	<0.01	<0.001	<0.001	<0.001	<0.001	<0.001	ns	<0.001	Ns
ALP (IU/L)	133 ± 16	136 ± 16	114 ± 9	136 ± 11	114 ± 5.7	121 ± 15	90 ± 9.5	122 ± 8.5	83 ± 7.8	120 ± 2.1	83 ± 5.7
*p* value		ns	<0.05	ns	<0.05	ns	<0.001	ns	<0.001	ns	<0.001

## Data Availability

The data presented in this study are available in the article.

## References

[1] Hornback N.B. (1989). Historical aspects of hyperthermia in cancer therapy. Radiol. Clin. N. Am..

[2] Seegenschmiedt M.H., Vernon C.C., Seegenschmiedt M.H., Fessenden P., Vernon C.C. (1995). A Historical Perspective on Hyperthermia in Oncology. Thermoradiotherapy and Thermochemotherapy: Biology, Physiology, Physics.

[3] LeBrun A., Zhu L., Shrivastava D. (2018). Magnetic Nanoparticle Hyperthermia in Cancer Treatment: History, Mechanism, Imaging-Assisted Protocol Design, and Challenges. Theory and Applications of Heat Transfer in Humans.

[4] Jordan A., Wust P., Fählin H., John W., Hinz A., Felix R. (1993). Inductive heating of ferrimagnetic particles and magnetic fluids: Physical evaluation of their potential for hyperthermia. Int. J. Hyperth..

[5] Jordan A., Wust P., Scholz R., Faehling H., Krause J., Felix R., Häfeli U., Schütt W., Teller J., Zborowski M. (1997). Magnetic Fluid Hyperthermia (MFH). Scientific and Clinical Applications of Magnetic Carriers.

[6] Jordan A., Scholz R., Wust P., Fähling H. (1999). Roland Felix Magnetic fluid hyperthermia (MFH): Cancer treatment with AC magnetic field induced excitation of biocompatible superparamagnetic nanoparticles. J. Magn. Magn. Mater..

[7] Périgo E.A., Hemery G., Sandre O., Ortega D., Garaio E., Plazaola F., Teran F.J. (2015). Fundamentals and advances in magnetic hyperthermia. Appl. Phys. Rev..

[8] Mornet S., Vasseur S., Grasset F., Duguet E. (2004). Magnetic nanoparticle design for medical diagnosis and therapy. J. Mater. Chem..

[9] Pankhurst Q.A., Connolly J., Jones S.K., Dobson J. (2003). Applications of magnetic nanoparticles in biomedicine. J. Phys. D. Appl. Phys..

[10] Dutz S., Hergt R. (2014). Magnetic particle hyperthermia—A promising tumour therapy?. Nanotechnology.

[11] Gilchrist R.K., Medal R., Shorey W.D., Hanselman R.C., Parrott J.C., Taylor C.B. (1957). Selective Inductive Heating of Lymph Nodes. Ann. Surg..

[12] Liu X., Zhang Y., Wang Y., Zhu W., Li G., Ma X., Zhang Y., Chen S., Tiwari S., Shi K. (2020). Comprehensive understanding of magnetic hyperthermia for improving antitumor therapeutic efficacy. Theranostics.

[13] Tay Z.W., Chandrasekharan P., Fellows B.D., Rodrigo Arrizabalaga I., Yu E., Olivo M., Conolly S.M. (2021). Magnetic Particle Imaging: An Emerging Modality with Prospects in Diagnosis, Targeting and Therapy of Cancer. Cancers.

[14] Atkinson W.J., Brezovich I.A., Chakraborty D.P. (1984). Usable Frequencies in Hyperthermia with Thermal Seeds. IEEE Trans. Biomed. Eng..

[15] Brezovich I.A. (1988). Low frequency hyperthermia: Capacitive and ferromagnetic thermoseed methods. Med. Phys. Monogr..

[16] Gneveckow U., Jordan A., Scholz R., Brüß V., Waldöfner N., Ricke J., Feussner A., Hildebrandt B., Rau B., Wust P. (2004). Description and characterization of the novel hyperthermia- and thermoablation-system for clinical magnetic fluid hyperthermia. Med. Phys..

[17] Mahmoudi K., Bouras A., Bozec D., Ivkov R., Hadjipanayis C. (2018). Magnetic hyperthermia therapy for the treatment of glioblastoma: A review of the therapy’s history, efficacy and application in humans. Int. J. Hyperth..

[18] Dhar D., Ghosh S., Das S., Chatterjee J. (2022). A review of recent advances in magnetic nanoparticle-based theranostics of glioblastoma. Nanomedicine.

[19] Hergt R., Dutz S. (2007). Magnetic particle hyperthermia-biophysical limitations of a visionary tumour therapy. J. Magn. Magn. Mater..

[20] Arriortua O.K., Garaio E., Herrero de la Parte B., Insausti M., Lezama L., Plazaola F., García J.A., Aizpurua J.M., Sagartzazu M., Irazola M. (2016). Antitumor magnetic hyperthermia induced by RGD-functionalized Fe_3_O_4_ nanoparticles, in an experimental model of colorectal liver metastases. Beilstein J. Nanotechnol..

[21] de la Parte B.H., Irazola M., Pérez-Muñoz J., Rodrigo I., Iturrizaga Correcher S., Mar Medina C., Castro K., Etxebarria N., Plazaola F., García J.Á. (2021). Biochemical and Metabolomic Changes after Electromagnetic Hyperthermia Exposure to Treat Colorectal Cancer Liver Implants in Rats. Nanomaterials.

[22] Dössel O., Bohnert J. (2013). Safety considerations for magnetic fields of 10 mT to 100 mT amplitude in the frequency range of 10 kHz to 100 kHz for magnetic particle imaging. Biomed. Technol..

[23] Kossatz S., Ludwig R., Dähring H., Ettelt V., Rimkus G., Marciello M., Salas G., Patel V., Teran F.J., Hilger I. (2014). High Therapeutic Efficiency of Magnetic Hyperthermia in Xenograft Models Achieved with Moderate Temperature Dosages in the Tumor Area. Pharm. Res..

[24] Thiesen B., Jordan A. (2008). Clinical applications of magnetic nanoparticles for hyperthermia. Int. J. Hyperth..

[25] Jordan A., Scholz R., Maier-Hauff K., van Landeghem F.K.H., Waldoefner N., Teichgraeber U., Pinkernelle J., Bruhn H., Neumann F., Thiesen B. (2006). The effect of thermotherapy using magnetic nanoparticles on rat malignant glioma. J. Neurooncol..

[26] Lee J.-H., Jang J., Choi J., Moon S.H., Noh S., Kim J., Kim J.-G., Kim I.-S., Park K.I., Cheon J. (2011). Exchange-coupled magnetic nanoparticles for efficient heat induction. Nat. Nanotechnol..

[27] Prabhu S., Mutalik S., Rai S., Udupa N., Rao B.S.S. (2015). PEGylation of superparamagnetic iron oxide nanoparticle for drug delivery applications with decreased toxicity: An in vivo study. J. Nanoparticle Res..

[28] Zhou X., Wang L., Xu Y., Du W., Cai X., Wang F., Ling Y., Chen H., Wang Z., Hu B. (2018). A pH and magnetic dual-response hydrogel for synergistic chemo-magnetic hyperthermia tumor therapy. RSC Adv..

[29] Yu K., Liang B., Zheng Y., Exner A., Kolios M., Xu T., Guo D., Cai X., Wang Z., Ran H. (2019). PMMA-Fe_3_O_4_ for internal mechanical support and magnetic thermal ablation of bone tumors. Theranostics.

[30] Soleymani M., Khalighfard S., Khodayari S., Khodayari H., Kalhori M.R., Hadjighassem M.R., Shaterabadi Z., Alizadeh A.M. (2020). Effects of multiple injections on the efficacy and cytotoxicity of folate-targeted magnetite nanoparticles as theranostic agents for MRI detection and magnetic hyperthermia therapy of tumor cells. Sci. Rep..

[31] Kulikov O.A., Zharkov M.N., Ageev V.P., Yakobson D.E., Shlyapkina V.I., Zaborovskiy A.V., Inchina V.I., Balykova L.A., Tishin A.M., Sukhorukov G.B. (2022). Magnetic Hyperthermia Nanoarchitectonics via Iron Oxide Nanoparticles Stabilised by Oleic Acid: Anti-Tumour Efficiency and Safety Evaluation in Animals with Transplanted Carcinoma. Int. J. Mol. Sci..

[32] Epstein Y., Yanovich R. (2019). Heatstroke. N. Engl. J. Med..

[33] Pan J., Xu Y., Wu Q., Hu P., Shi J. (2021). Mild Magnetic Hyperthermia-Activated Innate Immunity for Liver Cancer Therapy. J. Am. Chem. Soc..

[34] Mustafa S., Elgazzar A.H., Essam H., Gopinath S., Mathew M. (2007). Hyperthermia Alters Kidney Function and Renal Scintigraphy. Am. J. Nephrol..

[35] Krafft J., Fink R., Rosalki S.B. (1977). Serum Enzymes and Isoenzymes after Surgery. Ann. Clin. Biochem..

[36] Graeber C.G.M., Clagett G.P., Wolf R.E., Cafferty P.J., Harmon C.J.W., Rich N.M. (1990). Alterations in Serum Creatine Kinase and Lactate Dehydrogenase: Association with Abdominal Aortic Surgery, Myocardial Infarction and Bowel Necrosis. Chest.

[37] Yousef M.A., Vaida S., Somri M., Mogilner J., Lanir A., Tamir A., Shaoul R. (2006). Changes in creatine phosphokinase (CK) concentrations after minor and major surgeries in children. BJA Br. J. Anaesth..

[38] Alzeer A.H., El-Hazmi M.A.F., Warsy A.S., Ansari Z.A., Yrkendi M.S. (1997). Serum enzymes in heat stroke: Prognostic implication. Clin. Chem..

[39] Yang M.-M., Wang L., Zhang Y., Yuan R., Zhao Y., Hu J., Zhou F.-H., Kang H.-J. (2020). Establishment and effectiveness evaluation of a scoring system for exertional heat stroke by retrospective analysis. Mil. Med. Res..

[40] Iba T., Connors J.M., Levi M., Levy J.H. (2022). Heatstroke-induced coagulopathy: Biomarkers, mechanistic insights, and patient management. eClinicalMedicine.

[41] Hashim I.A. (2010). Clinical biochemistry of hyperthermia. Ann. Clin. Biochem..

[42] Nie J., Tong T.K., George K., Fu F.H., Lin H., Shi Q. (2011). Resting and post-exercise serum biomarkers of cardiac and skeletal muscle damage in adolescent runners. Scand. J. Med. Sci. Sports.

[43] Brancaccio P., Maffulli N., Buonauro R., Limongelli F.M. (2008). Serum Enzyme Monitoring in Sports Medicine. Clin. Sports Med..

[44] Weibrecht K., Dayno M., Darling C., Bird S.B. (2010). Liver Aminotransferases Are Elevated with Rhabdomyolysis in the Absence of Significant Liver Injury. J. Med. Toxicol..

[45] Lim A.K.H., Arumugananthan C., Lau Hing Yim C., Jellie L.J., Wong E.W.W., Junckerstorff R.K. (2020). A Cross-Sectional Study of the Relationship between Serum Creatine Kinase and Liver Biochemistry in Patients with Rhabdomyolysis. J. Clin. Med..

[46] Lim A.K. (2020). Abnormal liver function tests associated with severe rhabdomyolysis. World J. Gastroenterol..

[47] Zhang X., Chen Y., Tang L., Zhang Y., Duan P., Su L., Tong H. (2018). The liver sinusoidal endothelial cell damage in rats caused by heatstroke. Eur. J. Inflamm..

[48] Li D., Wang X., Liu B., Liu Y., Zeng Z., Lu L., Zheng Z., Li B., Zheng Z. (2015). Exercises in Hot and Humid Environment Caused Liver Injury in a Rat Model. PLoS ONE.

[49] Ward M.D., King M.A., Gabrial C., Kenefick R.W., Leon L.R. (2020). Biochemical recovery from exertional heat stroke follows a 16-day time course. PLoS ONE.

[50] Collins C.M. (2016). Tissue/field interactions, MRI safety, and field-related image artifacts. Electromagnetics in Magnetic Resonance Imaging: Physical Principles, Related Applications, and Ongoing Developments.

